# Developmental neurotoxicity of an anatoxin-a-producing cyanobacteria (*Aphanizomenon gracile*) lysate to zebrafish (*Danio rerio*)

**DOI:** 10.3389/fmicb.2025.1623621

**Published:** 2025-09-09

**Authors:** Chang Liu, Baiyu Cui, Lian Hu, Rui Li, Peng Xiao, Jun Zuo, Zeshuang Wang, Zengling Ma, Yuping Fan, He Zhang, Renhui Li

**Affiliations:** ^1^Zhejiang Provincial Key Laboratory for Subtropical Water Environment and Marine Biological Resources Protection, National and Local Joint Engineering Research Center of Ecological Treatment Technology for Urban Water Pollution, College of Life and Environmental Sciences, Wenzhou University, Wenzhou, China; ^2^Wenzhou Shanxi Hydro-junction Management Center, Wenzhou, China; ^3^Wenzhou Wencheng Ecological Environmental Monitoring Station, Wenzhou, China

**Keywords:** harmful cyanobacterial bloom, cyanotoxin, developmental neurotoxicity, oxidative stress, apoptosis

## Abstract

Harmful cyanobacterial blooms occur frequently worldwide with serious environmental impacts. Many detrimental cyanobacteria are well known for their potential to synthesize various cyanotoxins, posing significant threats to aquatic ecosystems and public health worldwide. While most studies focus on the toxicological impacts of microcystins and its main producer *Microcystis aeruginosa*, the ecotoxic effects of anatoxin-a and anatoxin-a-producing cyanobacteria are not fully understood. This study investigated the response of zebrafish (*Danio rerio*) to a ruptured cell solution (RCS) of a planktonic anatoxin-a-producing *Aphanizomenon gracile*. The RCS exposure negatively affected the hatching of zebrafish embryos, and promoted malformation. Furthermore, RCS treatment also disrupted neurobehaviors, and induced severe oxidative stress. In addition, the RCS inhibited the activity of acetylcholinesterase, and dysregulated the expression of several genes related to neuron-development and activated apoptosis in zebrafish. These results suggest that blooms of anatoxin-a-producing *Aphanizomenon gracile* will have neurotoxic effects on aquatic animals, and could impact human health. They help to further understand the potential neurotoxicity of harmful cyanobacteria, and highlight the need for further studies and risk assessments of the ecological impacts of harmful cyanobacterial blooms in freshwater ecosystems.

## Graphical abstract

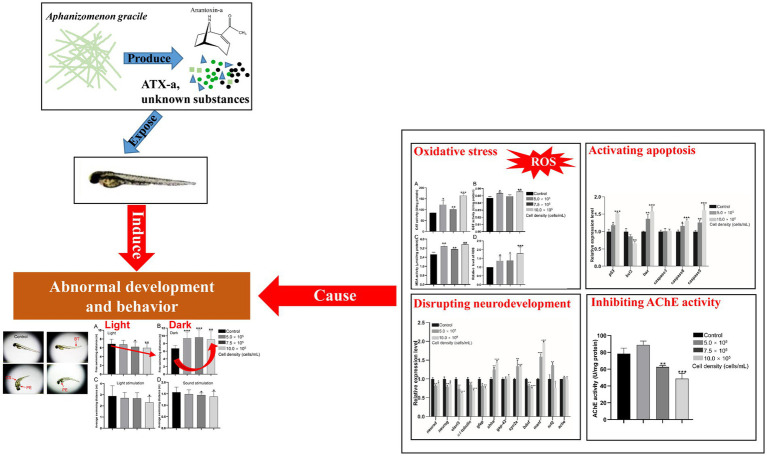

## 1 Introduction

In recent years, the occurrence and duration of harmful cyanobacterial blooms (cyanoHABs) have progressively increased worldwide, posing significant threats to both ecosystems and public health (Huisman et al., 2018; Huo et al., 2021). Beside the well-known dominant species *Microcystis* and *Raphidiopsis*, *Aphanizomenon* spp. also forms cyanoHABs in freshwater bodies (Cirés and Ballot, 2016), including in China (Liu et al., 2006; Wang et al., 2015), and USA (Burdick et al., 2020). Moreover, *Aphanizomenon gracile* (*A. gracile*) has been one of the most commonly detected species (Wang et al., 2015; Cirés and Ballot, 2016). *Aphanizomenon* blooms also contribute to the production of neurotoxins anatoxins [e.g., anatoxin-a; 2-acetyl-9-azabycyclo (4.2.1.) non-2-ene (ATX-a)] and paralytic shellfish-poisoning toxins (e.g., saxitoxins) (Wang et al., 2015; Cirés and Ballot, 2016), threatening aquatic organisms and public health. However, the potential acute toxicity of harmful *Aphanizomenon* blooms has not been fully studied, especially compared with that of *Microcystis* spp. and *Raphidiopsis* spp.

Anatoxins (including ATX-a) have been frequently detected in aquatic ecosystems across the European, Asia–Pacific, and North American regions (Lovin and Brooks, 2020; Christensen and Khan, 2020). A meta-analysis of published data from 1988 to 2018 indicated that concentrations of ATX-a of >0.1, 1.0, and 300 μg/L in 79.62, 48.37, and 1.42% of water bodies respectively, and the highest concentration of 172, 640 μg/L was reported in Lake Anderson (USA) (Lovin and Brooks, 2020). ATX-a is an extreme toxic toxin that is also known as the “very fast death factor” (Devlin et al., 1977; Colas et al., 2021). It can irreversibly bind to nicotinic acetylcholine receptors (nAChRs), and block normal neurotransmission between neurons and muscles, resulting in muscular fasciculation, loss of coordination, and respiratory paralysis; for example, intraperitoneal injection resulted in the death of mice 2–5 min after administration (LD_50_ = 200 mg/kg) (Devlin et al., 1977; Colas et al., 2021; Christensen and Khan, 2020). Several poisoning events have been reported, including in humans, dogs, and birds (Cirés and Ballot, 2016; Colas et al., 2021; Christensen and Khan, 2020), thereby ATX-a and its potential producers pose a great risk to both aquatic environments and human health. However, the toxic effects of ATX-a varied in different laboratories, and even exhibited remarkable discrepancies. Romero-Alfano et al. (2024) found that low concentrations of ATX-a induced hypolocomotion in zebrafish larvae after a 24 h exposure, while no significant photolocomotor behavioral changes were observed when exposed to 11–3,490 μg/L of ATX-a (Lovin et al., 2021). Similar less toxicity results also observed in Neuro2a cell lines (Takser et al., 2016), and several other studies (Plata-Calzado et al., 2022). Therefore, the neurotoxic effects of ATX-a need additional research.

Furthermore, an increasing number of studies report that cell-free lysates from cyanotoxin-producing cyanobacteria induced more severe toxic effects compared with pure cyanotoxin alone (Oberemm et al., 1997; Le Manach et al., 2016; Saraf et al., 2018), suggesting that the mixture of cyanobacterial metabolites might work synergistically with cyanotoxins on aquatic animals. Osswald et al. (2009) also reported that cyanobacterial extracts from ATX-a producing *Anabaena* sp. (ANA 37) posed more harm to carp (*Cyprinus carpio*) at early developmental stages, when compared that of pure ATX-a. Extracts from benthic ATX-a producing *Phormidium* spp. also exhibited strong toxicity to 3 macroinvertebrates (Anderson et al., 2018). However, the intoxication effects and underlying mechanisms of ATX-a containing lysates are largely unknown, and further investigation is urgently needed. Thus, the aquatic animal model zebrafish is used to study the developmental neurotoxicity of the lysate from an ATX-a-producing *A. gracile* from individual and molecular levels. Our results will help bridge the current knowledge gap, and provide more novel insights into the developmental neurotoxicity of harmful cyanobacteria, helping to evaluate the health risks of cyanoHABs and to manage the safety of aquatic ecosystems.

## 2 Materials and methods

### 2.1 Cyanobacterial cultures

ATX-a producing cyanobacteria *A. gracile* CHAB-1039 was obtained from the Institute of Hydrobiology of the Chinese Academy of Science (Wuhan, China), and kept in our laboratory. It was cultured in sterilized CT medium at temperature 25 ± 0.5 °C with an initial cell density of 5 × 10^3^ cells/mL under white fluorescent lights (40 μmol m^−2^ s^−1^, 12 h light/12 h dark cycle). Cell density of filamentous cyanobacteria was determined by a phytoplankton chamber method (Xie et al., 2024). Cyanobacterial ruptured cell solution (RCS) were prepared as previously described (Niu et al., 2021; Lu et al., 2023), and the details and used regents were provided in Supplementary materials.

### 2.2 Experimental design

Zebrafish has been a general subject for toxicity tests, and the fertilized eggs (2 h-post-fertilization) are selected for *A. gracile* RCS toxicity in the present study. Staging of zebrafish embryos followed previous studies (Kimmel et al., 1995). At 24 h-post-fertilization (hpf), the embryo has developed into a heart, a tail and two eyes. At 72 hpf, the embryo hatch and has developed into larvae with various organ rudiments. The larvae would develop well within 120 hpf. They are convenient for neuro-behavior analysis for developed visual and motor systems (Colwill, 2019; Qian et al., 2018). Considering aquatic embryos can hatch prematurely for external stressors (Cowan et al., 2024), the hatching rate was recorded from 24 hpf. The following developmental parameters, including heartbeat, malformation, and survival rate, biochemical indicators (i.e., reactive oxygen species, and malondialdehyde content), gene transcriptional level, and locomotor behavior were evaluated at 120 hpf as a previous study described (Niu et al., 2021). Preliminary experiments showed that *A. gracile* RCS with low abundance (5.0 × 10^4^ cells/mL) did not cause significant toxicity on zebrafish embryos underwent acute exposure, while most larvae were dead when treated with high concentrations (1.0 × 10^7^ cells/mL) (data not shown). Osswald et al. (2007) also reported similar results when studied the toxicity of an ATX-a producing *Anabaena* sp. at sub-lethal concentrations. Thereby, three sub-lethal concentration gradient intervals (5.0 × 10^5^, 7.5 × 10^5^, and 10.0 × 10^5^ cells/mL) were chosen to study the non-lethal effects in the following laboratory assays.

### 2.3 Zebrafish maintenance and developmental toxicity tests

Wild-type adult zebrafish (AB strain) were from the China Zebrafish Resource Center (Institute of Hydrobiology of the Chinese Academy of Science, Wuhan, China), and maintained in a fish incubation system (Z-A-D5, Haisheng Biotech CO., LTD, Shanghai, China) at 28 ± 0.5 °C. Procedures of zebrafish mating, spawning and embryo maintenance were followed methods described in previous references (Niu et al., 2021; Su et al., 2023). Fertilized eggs were collected, and selected under a stereomicroscope within 2 hpf. Embryos with normal morphology were rinsed with embryo medium, and used for the following toxicity experiments.

Experimental embryos (approximately 75 each group) were exposed to freshly prepared RCS of *A. gracile* CHAB-1039 solutions (5.0 × 10^5^, 7.5 × 10^5^, 10.0 × 10^5^ cells/mL) at 28.5 ± 0.5 °C from 6 to 120 hpf. The control group was cultured in EM without RCS. Half of the exposure medium was renewed every 24 h, and the dead embryos were recorded and removed simultaneously. All experiments were performed in triplicate. The morphological changes were observed and photographed using a stereomicroscope (Olympus, Japan). The survival rate, malformation rate, hatchability, and heat beat were recorded at indicated time points. Finally, zebrafish larvae were collected for biochemical and other experiments. All experiments conducted on animals were approved by the Institutional Review Board of Wenzhou University.

### 2.4 Locomotor behavior analysis

The locomotor behavior of zebrafish larvae was analyzed by a Zebralab high-throughput Video-Track system (ViewPoint Life Sciences, France) according to two previous studies (Niu et al., 2021; Su et al., 2023). Briefly, ten living larvae at 120 hpf without deformity was randomly selected from each group (3 replicates for a total of 30 larvae per treatment), and then transferred to a 96-well plate (length × width: 1270 mm × 860 mm, well diameter 75 mm; one fish per well) with a 300 μL of E3 medium per well. Zebrafish larvae were acclimated to the environment for 20 min (dark, 28 °C), and the high-throughput monitor system was set in tracking mode before behavior monitoring. Then, the locomotor behavior was recorded under indicated conditions. Finally, the data were exported from the high-throughput monitor system, and used to analyze the free swimming distance and average swimming distance under different treatment. The details of experimental method were provided in Supplementary material.

### 2.5 Oxidative stress analysis

After 120 h exposure, about 30 larvae fish were randomly collected from each group, and homogenized in cold phosphate buffered saline (PBS, pH 7.4) with protease inhibitor cocktails. Homogenate was centrifuged (4 °C, 10 min, at 12, 000 rpm). Obtained supernatant was used for detection of activities of catalase (CAT), glutathione S-transferase (GST), and the content of malondialdehyde (MDA). The reactive oxygen species (ROS) level was determined using the 2′,7′-dichlorodihydrofluorescein diacetate (DCFH-DA) probe following the manufacturer’s protocol and previous studies (Zhou et al., 2021). The details of experimental method were provided in Supplementary materials.

### 2.6 Acetylcholinesterase activity analysis

After 120 h of cyanobacterial lysate exposure, approximately 30 larvae from each replicate (*n* = 3) were randomly collected, and homogenized in 500 μL of cold phosphate buffer saline on ice. Then the supernatant was obtained via centrifugation (4 °C, 10 min, 12, 000 rpm), and the protein concentration was determined. Then the acetylcholinesterase (AChE) activity was monitored using an AChE assay kit according to the manufacturer’s instruction and two previous studies (Guo et al., 2018; Niu et al., 2021).

### 2.7 Quantitative real-time PCR

qRT-PCR assay was carried out as Su et al. (2023) described. Gene description and primers used in this study are provided in Supplementary material. Because the preliminary results (i.e., locomotion behavior and AChE activity) of the 7.5 × 10^5^, and 10.0 × 10^5^ cells/mL treatments were similar, but different from that of 5.0 × 10^5^cells/mL treatment group. Thereby, we analyzed gene expression level and green fluorescent protein (GFP) intensity in transgenic zebrafish at concentrations of 0, 5.0 × 10^5^ cells/mL, and 10.0 × 10^5^ cells/mL in the subsequent assays. Briefly, total RNA extracted from thirty zebrafish larvae after exposure with Trizol Reagent, and the concentration and quality was determined using a Nanodrop 2000 (Thermo Scientific, United States). A Prime Script™ RT reagent kit with gDNA Eraser was used to eliminate genomic DNA, and synthesize cDNA according to the manufacturer’s protocol. All qRT-PCR reactions were conducted on a 96-well plate format in QuantStudio1 (Thermo Scientific, United States) with a total volume of 20 μL containing 10 μL of Power SYBR® Green PCR master mixture. Twenty nanogram of cDNA was used as a template per well. The PCR program was as following: 50 °C for 2 min, then 95 °C for 10 min, following by 40 cycles (95 °C for 10 s, and 60 °C for 1 min). Melting curves and gel electrophoresis of amplification products were used to evaluate the reaction specificity, and the relative mRNA expression level of target genes was determined by the 2^−ΔΔCt^ method (Livak and Schmittgen, 2001).

### 2.8 Transgenic zebrafish larvae assay

Adult transgenic zebrafish (HuC green fluorescent protein, HuC:GFP, AB strain) were also obtained from the China Zebrafish Resource Center at Institute of Hydrobiology (Chinese Academy of Science, Wuhan, China). The maintenance of adult fish, and embryo exposure assay followed the protocols described in Section 2.3. After120-h treatment of various concentrations of cyanobacterial RCS (0, 5.0 × 10^5^, 10.0 × 10^5^ cells/mL), images of the Tg (HuC:GFP) larvae (3 replicates with 10 larvae per replicate) were obtained using a phase-contrast fluorescence microscope (LEICA DMi8, Germany). The GFP expression intensity was analyzed with the Image J software (http://rsbweb.nih.gov/ij, National Institutes of Health, United States) as Guo et al. (2018) and Gyimah et al. (2021) described.

### 2.9 Statistical analysis

All assays were performed in triplicate and data are presented as the mean ± standard deviation (SD). Statistical analysis was conducted with GraphPad Prism V8.0 (GraphPad software). After assessment of normality and homogeneity of variance, the differences between the control and each treated group were evaluated by one-sample *T*-test or a one-way analysis of variance with the statistical software SPSS Statistics 21. Statistical significance was set at *p* < 0.05, and * *p* < 0.05, ** *p* < 0.01, and *** *p* < 0.001.

## 3 Results

### 3.1 Effects of RCS on embryonic development

**Figure 1:**
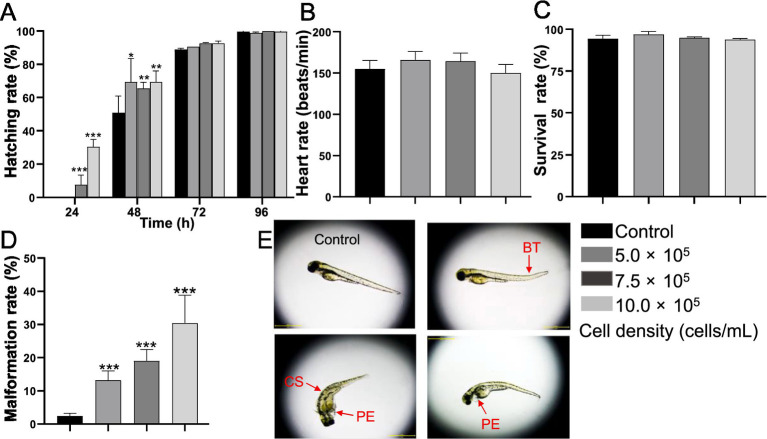
Developmental toxicity induced by *A. gracile* RCS in zebrafish embryos at 120 hpf. **(A)** Hatching rate. **(B)** Heartbeat. **(C)** Survival rate. **(D)** Malformation rate. **(E)** Morphology of zebrafish larvae exposed to RCS. BT, bent tail; PE, pericardial edema; CS, curved spine. The values are expressed as mean ± SD (*n* = 3, 20 larvae/replicate). * *p* < 0.05, ** *p* < 0.01, *** *p* < 0.001.

The hatching rate was significantly increased by *A. gracile* RCS from the early exposure phase. At 24 hpf, the hatching rate was 7.65% (*p* < 0.001) and 30.32% (*p* < 0.001) for the 7.5 × 10^5^ and 10.0 × 10^5^ cells mL^−1^groups, respectively, whereas no larvae hatched in the control group (Figure 1A). At 48 hpf, the hatchability increased to 69.34% (*p* < 0.05), 65.55% (*p* < 0.01), and 69.41% (*p* < 0.01) in the 5 × 10^5^, 7.5 × 10^5^, and 10.0 × 10^5^ cells mL^−1^ treated groups respectively, which were much higher than that of the control group (50.91%). While no significant effect was observed among the RCS treatment groups and the control group at 72 and 96 hpf (*p* > 0.05). Moreover, there was no statistical difference in heartbeat between each group at 120 hpf (Figure 1B). However, *A. gracile* RCS exposure caused obvious malformations in the embryos (e.g., bent tail, curved spine, and pericardial edema) in a dose-dependent manner (Figures 1D,E). The malformation rate was 13.13% (*p* < 0.001), 19.02% (*p* < 0.001), and 30.44% (*p* < 0.001) in the 5 × 10^5^, 7.5 × 10^5^, and 10.0 × 10^5^ cells/mL groups at 120 hpf (Figure 1D), respectively, when compared with the control group (2.35%). However, *A. gracile* RCS treatment at any concentration did not affect the survival rate at 120 hpf (*p* > 0.05) (Figure 1C).

### 3.2 Effects of RCS on the locomotor behavior of zebrafish larvae

**Figure 2:**
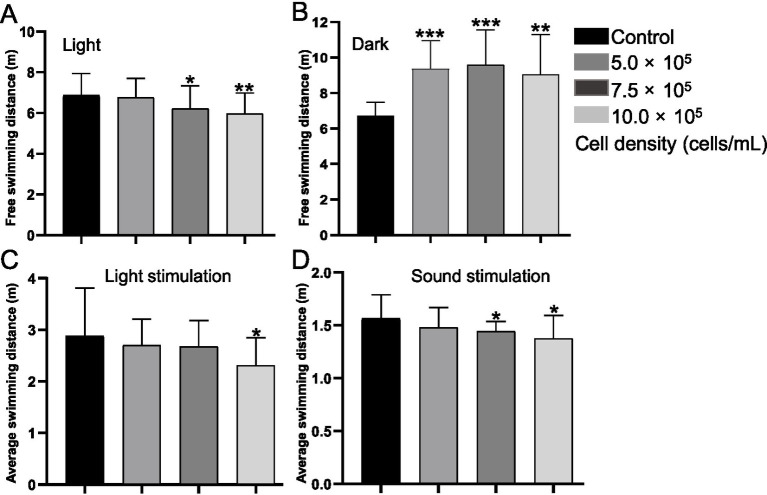
Behavior tests of *A. gracile* RCS treated zebrafish larvae. Zebrafish larvae exposed to *A. gracile* RCS at 120 hpf was assessed. **(A)** Average swimming distance under light condition. **(B)** Average swimming distance under dark condition. **(C)** Average swimming distance under 100% light stimulation. **(D)** Average swimming distance under sound stimulation. The values are expressed as mean ± SD (*n* = 3, 15 larvae/replicate). * *p* < 0.05, ** *p* < 0.01, *** *p* < 0.001.

Compared with the control group, the average swimming distance in response to light decreased by 2.28% (*p* > 0.05), 11.07% (*p* < 0.05) and 12.87% (*p* < 0.01) in the 5.0 × 10^5^, 7.5 × 10^5^ and 10.0 × 10^5^ cells/mL treatment groups, respectively (Figure 2A). By contrast, the swimming distance in the dark condition increased by 39.79% (*p* < 0.001), 42.96% (*p* < 0.001), and 35.01% (*p* < 0.001) in the 5.0 × 10^5^, 7.5 × 10^5^ and 10.0 × 10^5^ cells/mL treatment groups (Figure 2B). Moreover, only the 10.0 × 10^5^ cells/mL treated group showed insensitivity to light stimulation, and the average swimming distance reduced by 19.84% (*p* < 0.05) (Figure 2C). While, both the 7.5 × 10^5^ and 10.0 × 10^5^ cells/mL treatment groups appeared insensitivity to the sound stimulation, and the average swimming distance reduced by 7.67% (*p* < 0.05) and 12.04% (*p* < 0.05), compared with the control group (Figure 2D). There was no statistical difference between the 5.0 × 10^5^ cells/mL treatment group and the control (*p* > 0.05).

### 3.3 Effects of RCS on the AChE activity

**Figure 3:**
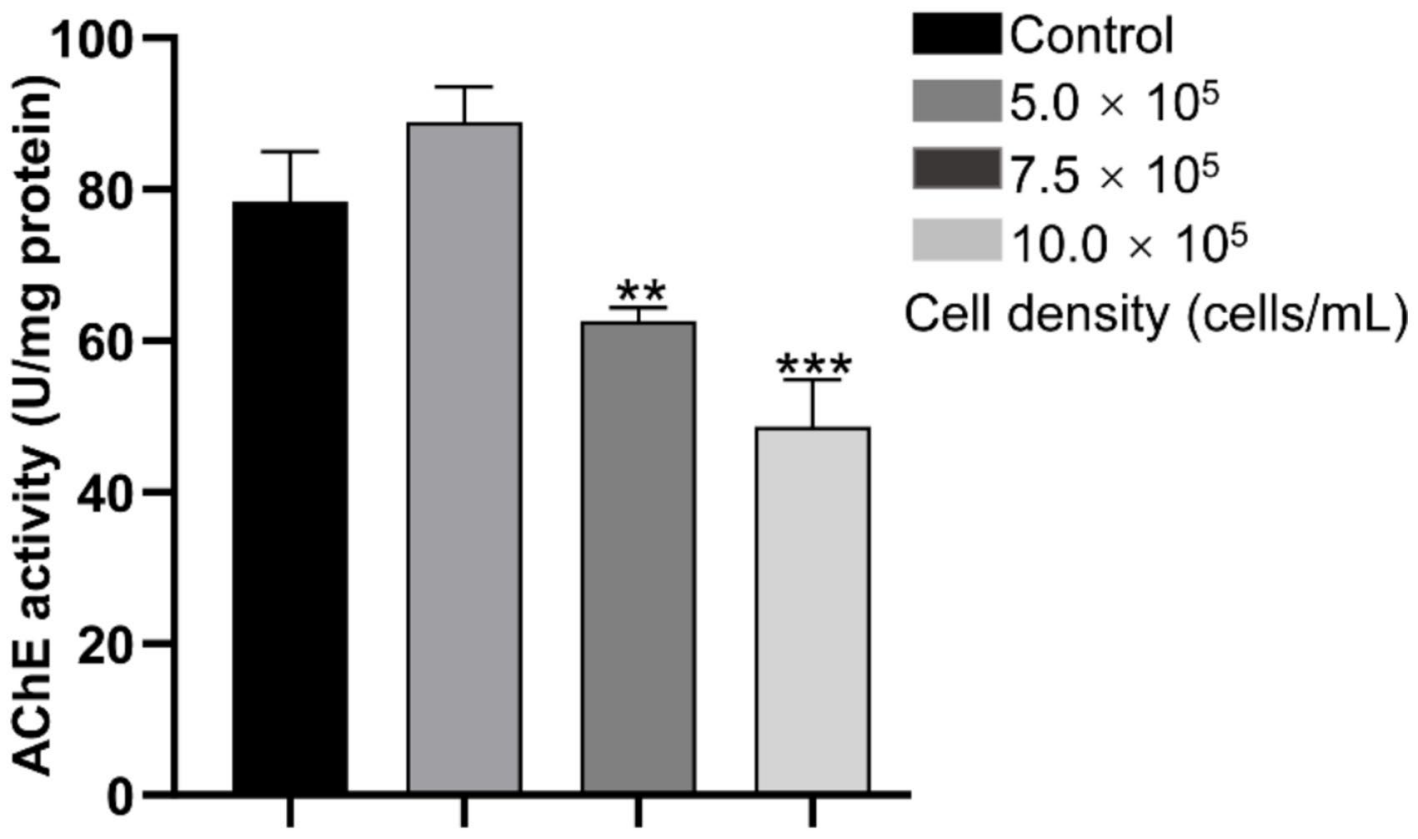
AChE activity of zebrafish larvae exposed to different concentrations of *A. gracile* RCS at 120 hpf. AChE activity. The values are expressed as mean ± SD (*n* = 3). ** *p* < 0.01, *** *p* < 0.001.

At 120 hpf, the AChE activity was significantly inhibited by 29.49% (*p* < 0.05), and 22.26% (*p* < 0.001) in the 7.5 × 10^5^ and 10.0 × 10^5^ cells/mL groups, respectively. Nevertheless, it increased 13.33% in the 5.0 × 10^5^ cells/mL group, but no significant difference was found when compared with the control group (*p* > 0.05) (Figure 3).

### 3.4 Effects of RCS on oxidative stress

**Figure 4:**
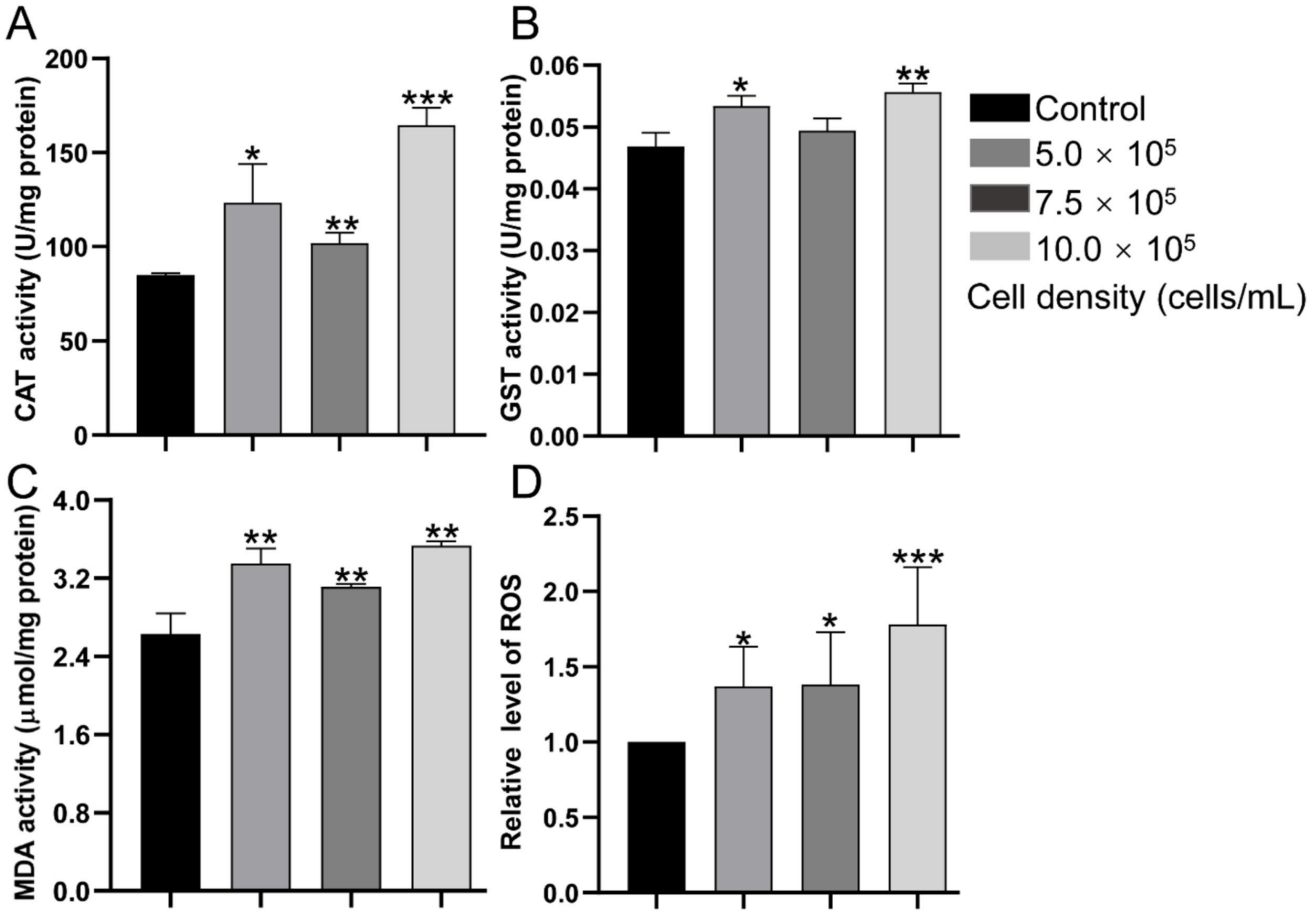
Redox response parameters of zebrafish larvae after exposed to *A. gracile* RCS at 120 hpf. **(A)** CAT activity. **(B)** GST activity. **(C)** MDA content. **(D)** Relative content of ROS. The values are expressed as mean ± SD (*n* = 3). * *p* < 0.05, ** *p* < 0.01, *** *p* < 0.001.

CAT activity reached to 123.26, 101.92, and 164.36 U/mg protein in the 5.0 × 10^5^, 7.5 × 10^5^, and 10.0 × 10^5^ cells/mL groups at 120 hpf respectively, and increased by 44.86% (*p* < 0.05), 19.78% (*p* < 0.01), and 93.16% (*p* < 0.001), compared with the control group (85.09 U/mg protein) (Figure 4A). RCS treatment also increased the activity of GST by 13.85% (*p* < 0.05), 5.51% (*p* > 0.05), and 18.80% (*p* < 0.01) in the 5.0 × 10^5^, 7.5 × 10^5^, and 10.0 × 10^5^ cells/mL groups, respectively (Figure 4B). In addition, the MDA content also significantly upregulated by 27.62% (*p* < 0.01), 18.55% (*p* < 0.01), and 34.68% (*p* < 0.01) in the 5.0 × 10^5^, 7.5 × 10^5^, and 10.0 × 10^5^ cells/mL groups, respectively (Figure 4C), in comparison to the control group (2.63 μmol/mg protein). RCS treatment also stimulated the production of ROS by 36.81% (*p* < 0.05), 38.27% (*p* < 0.05), and 78.04% (*p* < 0.001) in the 5.0 × 10^5^, 7.5 × 10^5^, and 10.0 × 10^5^ cells/mL groups, respectively, relative to the control group (Figure 4D).

### 3.5 Effects of RCS on the expression of apoptosis-related genes

**Figure 5:**
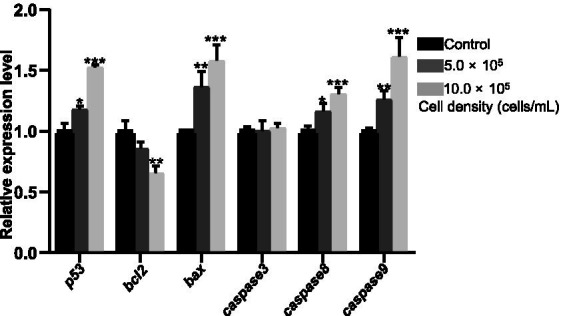
Relative expression levels of apoptotic-related genes in zebrafish larvae at 120 hpf after exposed to *A. gracile* RCS. The values are expressed as mean ± SD (*n* = 3). * *p* < 0.05, ** *p* < 0.01, *** *p* < 0.001.

The pro-apoptotic genes *p53* and *bax* were upregulated by 17.90% (*p* < 0.05) and 36.33% (*p* < 0.01) in the 5.0 × 10^5^ cells/mL group, and by 52.19% (*p* < 0.001) and 58.04% (*p* < 0.001) in the 10.0 × 10^5^ cells/mL group at 120 hpf, respectively, compared to the control group (Figure 5). By contrast, the anti-apoptotic gene *bcl2* was downregulated by 14.11% (*p* > 0.05) and 34.49% (*p* < 0.01) in the 5.0 × 10^5^ and 10.0 × 10^5^ cells/mL groups respectively, relative to the control group (Figure 5). Moreover, the transcription levels of *caspase 8* and *caspase 9* were enhanced by 16.35% (*p* < 0.05) and 25.91% (*p* < 0.01) in the 5.0 × 10^5^ cells/mL group, and by 30.45% (*p* < 0.001) and 61.35% (*p* < 0.001) in the 10.0 × 10^5^ cells/mL group, respectively (Figure 5), relative to the control group. However, the mRNA level of *caspase 3* did not change in any group (*p* > 0.05) (Figure 5).

### 3.6 Effects of RCS on the expression of genes associated with neurodevelopment

**Figure 6:**
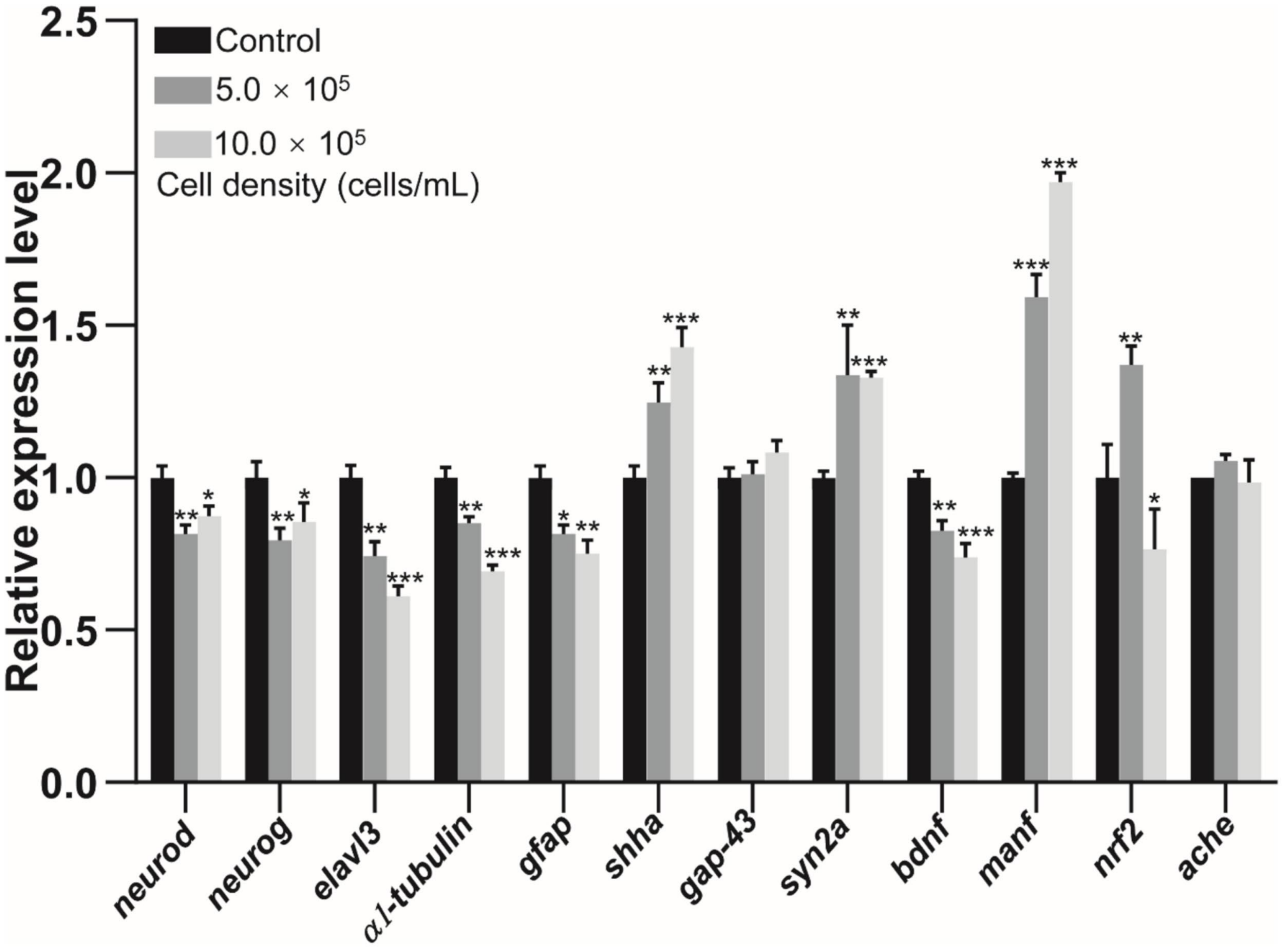
Relative expression levels of neurodevelopment-related genes in zebrafish larvae at 120 hpf after exposed to *A. gracile* RCS. The values are expressed as mean ± SD (*n* = 3). * *p* < 0.05, ** *p* < 0.01, *** *p* < 0.001.

The transcriptional levels of *neurod* and *neurog* were significantly downregulated by 18.36% (*p* < 0.01) and 20.57% (*p* < 0.01) in the 5.0 × 10^5^ cells/mL group, and by 12.57% (*p* < 0.05) and 14.39% (*p* < 0.05) in the 10.0 × 10^5^ cells/mL group at 120 hpf (Figure 6), when compared to the control group. The expression levels of *elavl3*, *α1-tubulin*, *gfap*, and *bdnf* reduced by 25.66% (*p* < 0.01), 14.94% (*p* < 0.01), 15.79% (*p* < 0.05), and 17.43% (*p* < 0.01) in the 5.0 × 10^5^cells/mL treated group, and by 38.80% (*p* < 0.001), 30.81% (*p* < 0.001), 24.89% (*p* < 0.01), and 26.17% (*p* < 0.001) in the 10.0 × 10^5^ cells/mL treatment group at 120 hpf respectively, compared with control group (Figure 6). By contrast, the transcription levels of *shha*, *syn2a*, and *manf* were upregulated by 24.69% (*p* < 0.01), 33.65% (*p* < 0.01), and 59.19% (*p* < 0.001) in the 5.0 × 10^5^ cells/mL group, and by 42.75% (*p* < 0.001), 32.85% (*p* < 0.001), and 96.90% (*p* < 0.001) in the 10.0 × 10^5^ cells/mL treatment group, relative to the control group. Moreover, the antioxidant gene *nrf2* induced by 36.94% (*p* < 0.01) in the 5.0 × 10^5^ cells/mL group, whereas it reduced by 23.34% (*p* < 0.05) in the 10.0 × 10^5^ cells/mL treatment group, compared with the control group (Figure 6). However, the transcription level of *gap-43* and *ache* did not significantly change in any treatment group (*p* > 0.05) (Figure 6).

### 3.7 Effects of RCS on neurogenesis in transgenic zebrafish larvae

**Figure 7:**
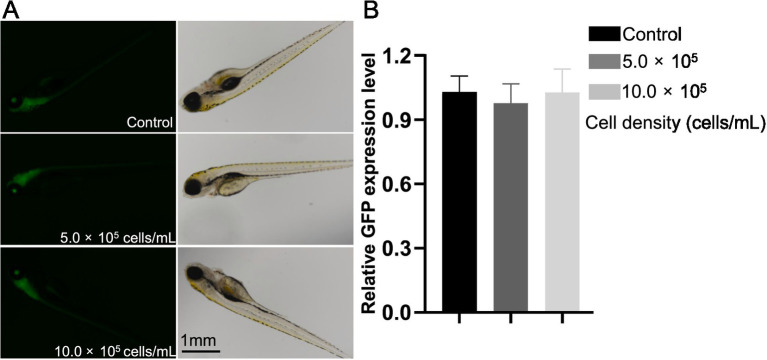
Effect of *A. gracile* RCS on Tg (HuC:GFP) zebrafish larvae at 120 hpf. **(A)** Representative images of the control and treated groups. **(B)** Relative GFP expression in larvae after exposed to *A. gracile* RCS. The values are expressed as mean ± SD (*n* = 3, 10 larvae/replicate).

A transgenic zebrafish line with neuron-specific GFP (HuC-GFP) was used to investigate the effects of cyanobacterial lysate on neurogenesis at early development stages. GFP fluorescence can observe in the brain of treated zebrafish (Figure 7A), but the intensity did not significantly change in any RCS treatment group at 120 hpf (*p* > 0.05) (Figure 7B), compared with the control group. The result suggested the Elavl3 level and neurogenesis did not abnormally change in the central nervous system after treatment.

## 4 Discussion

Blooms of *Aphanizomenon* spp. are frequently reported in freshwater bodies, resulting in several poisoning events with deaths of wildlife and animals, even humans (Cirés and Ballot, 2016; Lovin and Brooks, 2020). While limited research have systematically evaluated the ecological risks of ATX-a containing lysates on fish from the individual, biochemical, and molecular levels. Most studies just reported the toxicity (i.e., LD_90_, and clinical observations) of cyanobacterial extracts to aquatic animals, including goldfish, brine shrimp, and carp (Carmichael and Biggs, 1978; Kiviranta et al., 1991; Osswald et al., 2007; Osswald et al., 2009), and all of these studies lacked further investigation of potential intoxication mechanisms. Thereby, the developing embryos of zebrafish are used to investigate the toxic effects of *A. gracile* RCS under the occurrence of cyanoHABs setting.

In the present study, *A. gracile* RCS exposure promoted obvious premature hatching at 24 and 48 hpf (Figure 1A), and aggravated abnormal morphology in a concentration-dependent manner in zebrafish larvae, even at lower cell density (5.0 × 10^6^ cells/mL) (Figure 1E). The data clearly indicated the developmental toxicity of *A. gracile* RCS (Figure 1), and were consistent with the abnormal development parameters (e.g., hatching, malformation, and mortality) on fish larvae caused by ATX-a containing cyanobacterial extracts (Oberemm et al., 1999; Osswald et al., 2009). Notably, the results also verified that cyanoHABs might be one of the important environmental stressors stimulating aquatic embryos hatch prematurely (Qian et al., 2018; Cowan et al., 2024). The smaller size and morphological abnormalities would also make fish more susceptible to predator in the natural waters, and possibly contributed to the disappearance and unsuccessful recovery of various endangered fish species globally.

Neuro-behavior is one important index to assess the neurotoxicity (Qian et al., 2018; Cai et al., 2022; Jacinavicius et al., 2023), and neuro-impairment related to *A. gracile* RCS has been less studied in fish. Herein, we found that the RCS exposure significantly altered locomotor activity and response in zebrafish larvae (Figure 2). Notably, RCS of 5.0 × 10^5^ and 10.0 × 10^5^ cells/L significantly decreased locomotor activity in light conditions (Figure 2A), while all concentrations of RCS remarkably increased the locomotor activity in dark conditions (Figure 2B). Likewise, early exposure to ATX-a or toxic cyanobacteria also led to abnormal neurobehavior in zebrafish larvae (Qian et al., 2018; Lovin et al., 2024). Interestingly, RCS treatment resulted in hyperactivity under dark in zebrafish larvae accompanying by AChE inhibition, oxidative stress and impairment of neurodevelopment herein. Similar effects also had been reported (Ogungbemi et al., 2020; Pípal et al., 2020; Yi et al., 2025). For instance, fipronil sulfone (125 μg/L) induced hyperactivity in zebrafish larvae accompanying by AChE inhibition, oxidative stress and block of GABAergic receptors (Ogungbemi et al., 2020). 6-PPDQ (2 μg/L) exposure led to hyperactivity by inhibition of neurodevelopment, brain damage, and disturbance of neurotransmission pathways (Yi et al., 2025). Additionally, cyanobacterial extracts from samples dominated by *A. klebahnii* also led to hyperactivity by disrupting the retinoid signaling pathway, and down-regulation of *bdnf* (Pípal et al., 2020). Thereby, zebrafish hyperactivity after RCS treatment in the present study might due to disruption of neurodevelopment and neurotransmission (i.e., the retinoid and GABAergic signaling pathways), and the exact details need more experiments to validate. Additionally, we found that the larvae treated by RCS at 10.0 × 10^5^ cells/L even became insensitive to light and sound stimulation (Figures 2C,D), in coincidence with the data of zebrafish exposed to toxic *Karenia mikimotoi* (Niu et al., 2021). The resultant abnormal neurobehavior might potentially reduce the evasion ability of fish to avoid various predators, and cause survival and reproductive problems for population in the wild.

Comparative analyses indicated that zebrafish and *Daphnia* just displayed hypoactivity when exposed to 13–50, 000 μg/L of ATX-a (Bownik and Pawlik-Skowronska, 2019; Romero-Alfano et al., 2024; Lovin et al., 2024), and the concentrations were much higher than that of ATX-a in *A. gracile* RCS (1.21 × 10^−2^–2.42 × 10^−2^ μg/L). Obviously, *A. gracile* RCS exhibited higher toxicity than that of comparable concentrations of pure ATX-a toxin. Previous studies had revealed cyanobacterial RCS contain various metabolites (i.e., retinoids, anabaenopeptins, lipopeptides, alkaloids, and derivatives) (Jones et al., 2021; Jacinavicius et al., 2023; Wang B. et al., 2024), beyond the known cyanotoxins, and which can have toxic effects (Papendorf et al., 1997; Osswald et al., 2009; Falfushynska et al., 2021; Jacinavicius et al., 2023). These cyanometabolites might synergistically work with cyanotoxins against fish, and contribute to the developmental neurotoxicity observed. Moreover, lots of neurotoxic compounds had been identified, including anabaenopeptins in non-toxic *A. gracile* (Papendorf et al., 1997), 363 neurotoxic compounds in *Microcystis aeruginosa* lysate (Zi et al., 2023). A database called CyanoMetDB even collected more than 2010 cyanobacterial metabolites (Jones et al., 2021). Hence, toxicological studies of pure toxin alone might underestimate the potential toxicity of cyanoHABs, and using cyanobacterial lysates to mimic real exposure conditions for risk assessments and water management is essential. However, the toxicological outcomes in the real aquatic environments might be more complex than studies in laboratory. Various environmental factors might affect the toxicity. For instance, ambient temperature elevated the chronic toxicity of microcystin-LR in aquatic animals via affecting the uptake of cyanotoxin (Zhang et al., 2011; Ji et al., 2013; Kim et al., 2014). Kim et al. (2014) reported that NO_3_-N enhanced the chronic toxicity of microcystin-LR to *Moina macrocopa*, whereas NH_3_ -N reduced its toxicity (Kim et al., 2014). Other common contaminants in the aquatic environment (i.e., heavy metal and polystyrene microplastics) could also exacerbate the toxicity caused by cyanotoxins (Wei et al., 2020; Lin et al., 2025).

AChE can regulate neurobehavioral activity via degrading the neurotransmitter acetylcholine, and becomes a sensitive biomarker for risk assessment of neurotoxicant (Qian et al., 2018; Niu et al., 2021; Zhou et al., 2021). In this study, AChE activity was dose-dependently inhibited by the RCS (Figure 3), which might led to acetylcholine accumulation in synapses, affecting cholinergic neurotransmission and resulting in behavior changes. In line with that, RCS exposure significantly decreased the free swimming distance in zebrafish larvae under light conditions, and caused hypoactivity under light and sound stimulation (Figure 2). Similarly, exposure to toxic *M. aeruginosa*, and *Karenia mikimotoi* also reduced AChE and locomotor activities simultaneously in zebrafish (Qian et al., 2018; Niu et al., 2021). Hence, *A. gracile* RCS might promote locomotor behavioral anomalies via decreasing AChE activity and disrupting the balance of the neurotransmitters in zebrafish.

Oxidative stress is another important underlying mechanism involved in neurotoxicity. For instance, cyanometabolite 2-methylisoborneol and toxic *Karenia mikimotoi* exposure induced oxidative stress in a similar way in zebrafish larvae, resulting in hypoactivity and neurotoxicity (Niu et al., 2021; Zhou et al., 2021). Similarly, *A. gracile* RCS exposure induced a significant increase of ROS and MDA (Figures 4C,D), and activated the antioxidant enzymes CAT and GST (Figures 4A,B). These findings demonstrated that *A. gracile* RCS causes severe oxidative stress in zebrafish larvae. Previous studies suggest that ROS and oxidative stress can activate apoptosis, and disrupt homeostasis of the central nervous system, resulting in brain damage and hypoactivity in fish (Qi et al., 2016; Zhou et al., 2021). Specifically, ATX-a exposure also activated apoptosis in murine neuronal Neuro2a cells accompanied by oxidative stress (Takser et al., 2016). Hence, we speculated that apoptosis also involved in the neurotoxicity of *A. gracile* RCS, and the expression of apoptosis-related genes were studied.

The anti-oncogene *p53* was dose dependently upregulated when exposed to *A. gracile* RCS (Figure 5), and the pro-apoptotic gene *bcl2* also decreased in the treated groups at 120 hpf. Nevertheless, the anti-apoptotic gene *bax* significantly enhanced (Figure 5). Thereby, the *bax*/*bcl2* ratio increased to 1.59 (*p* < 0.001) and 2.43 (*p* < 0.001) respectively, which can activate apoptosis (Tsujimoto, 2003). In line with that, genes *caspase 8* and *caspase 9*, encoding apoptosis executors, remarkably enhanced in the 5 × 10^5^ and 10.0 × 10^5^ cells/L treated groups (Figure 5). Obvious apoptotic cells also observed in treated larvae using acridine orange staining (data not shown). *A. gracile* RCS-induced apoptosis might partially underlie the mechanism of neurotoxicity in zebrafish embryos. However, the cause and effect relationship should be validated by more experiments, including evaluation the change of Caspase activity, and rescue assay with different Caspase inhibitors.

Previous study reported that *M. aeruginosa* exposure downregulated *elavl3*, *gfap*, *neurog*, *manf* and *syn2a*, and decreased the locomotor activity in zebrafish larvae (Qian et al., 2018). The dysregulation of neurodevelopment genes might also involve in developmental neurotoxicity of *A. gracile* RCS. Hence, we examined the expression of these genes, and found that *neurod*, *neurog*, *gfap*, *elavl3*, α1*-tubulin*, and *bdnf* were downregulated to different degrees in the treated groups (Figure 6). The data implied that the neurodevelopment (e.g., neuronal differentiation and migration) of zebrafish embryos might negatively influence by *A. gracile* RCS, which contributed to the abnormal locomotor pattern (Figure 2). Additionally, *syn2a*, *shha* and *manf* were induced by *A. gracile* RCS exposure (Figure 6). Considering roles in neural plasticity and neuroprotection (Chen et al., 2012; Kao et al., 1998), their upregulation might be due to compensatory effects of zebrafish to fight against damage induced by exogenous contaminants, and verified the neurotoxicity of *A. gracile* RCS again. Taken together, disturbance of the expression of neurodevelopment genes might play important roles in the abnormal neuro-behavior in zebrafish larvae exposed to *A. gracile* RCS.

The adverse effects of *A. gracile* RCS on neurogenesis and Elavl3 protein level were further evaluated using a transgenic zebrafish (HuC:GFP) (Kim et al., 1996). Unexpectedly, no significant reduction in GFP intensity was observed in RCS-treated larvae (Figure 7), which was inconsistent with the decrease of *elavl3* mRNA (Figure 6). Similar discrepancies had also observed in zebrafish (Guo et al., 2018; Gyimah et al., 2021). This suggested that RCS might not work via affecting the Elavl3 expression and neurogenesis in zebrafish during short-time exposure. But the data could not exclude the developmental neurotoxicity of *A. gracile* RCS and those neurotoxicants in references (Guo et al., 2018; Gyimah et al., 2021): (1) Elavl3 and neurogenesis just two common biomarkers of those studies evaluated, and they could not represent the change tendency of other biomarkers, such as AChE activity; (2) dysfunctions of neural communication, synaptic plasticity and neural network in brain might also contribute to the developmental neurotoxicity without change the Elavl3 protein level, or neurogenesis (Frank et al., 2017; Ma et al., 2023). The exact mechanisms are still largely unknown, and need more studies.

Overall, the data herein document the potential neurotoxicity of harmful *A. gracile*, and highlight the adverse impacts of blooms forming cyanobacteria beside the dominant species *Microcystis* and *Raphidiopsis*. The present study also improves our understanding of the potential mechanisms of *A. gracile* on the early development of aquatic organisms. But some limitations are still needed to improve in the future study. Comparable concentration of pure ATX-a should be used in the positive control group, and the data can directly use to evaluate the toxic effects of RCS to avoid those obvious discrepancies in previous studies (Rogers et al., 2005; Osswald et al., 2009). Those unknown compounds beside cyanotoxins in the RCS might also involve in the neurotoxicity, and greatly hinder further mechanistic study. They could be identified using metabolomics, and screened *in silico* with public databases like CyanoMetDB (Jones et al., 2021), and artificial intelligence-powered PyRMD2Dock tool (Roggia et al., 2024). Their possible synergistic toxicity with ATX-a could be furtherly explored in laboratory via artificial intelligence-enabled zebrafish high-throughput screening systems (Wang N. et al., 2024). Specifically, more cultured fish species (i.e., gibel carp, and tilapia) should be used to evaluate the diverse toxicity to exclude the species-specific effects.

## 5 Conclusion

In summary, this study used *A. gracile* RCS to mimic a natural aquatic environment with cyanoHABs, and demonstrated that exposure to *A. gracile* RCS containing ATX-a promoted morphological abnormalities and exacerbated developmental neurotoxicity in zebrafish larvae. The neurodevelopmental toxicity in zebrafish was associated with oxidative stress, suppression of AChE activity, and dysregulation of neurodevelopment genes and activation of apoptosis. These results advance our understanding of the toxic effects and mechanisms of cyanoHABs, and emphasize the importance of using cyanobacterial lysate to assess the ecological threat and public health risks of cyanoHABs in future studies.

## Data Availability

The raw data supporting the conclusions of this article will be made available by the authors, without undue reservation.
