# Supplementary material for: Developmental neurotoxicity of an anatoxin-a-producing cyanobacteria (Aphanizomenon gracile) lysate to zebrafish (Danio rerio)
Source: Front Microbiol. 2025 Sep 9;16:1623621. doi: 10.3389/fmicb.2025.1623621 (PMC12454358; doi:10.3389/fmicb.2025.1623621)
Supplement: Supplementary file 1 [file Supplementary_file_1.docx]

**Supplementary Materials**

**Text S1-6, Table S1, and Figure S1**

**Developmental neurotoxicity of an anatoxin-a-producing cyanobacteria (*Aphanizomenon gracile*) lysate to zebrafish (*Danio rerio*)**

Chang Liu^a,1^, Baiyu Cui^b,1^, Lian Hu ^c,1^, Rui Li^a^, Peng Xiao^a^, Jun Zuo^a^, Zeshuang Wang^a^, Zengling Ma^a^, Yuping Fan ^a, *^, He Zhang^a, **^, Renhui Li^a^

^a^ Zhejiang Provincial Key Laboratory for Subtropical Water Environment and Marine Biological Resources Protection, National and Local Joint Engineering Research Center of Ecological Treatment Technology for Urban Water Pollution, College of Life and Environmental Sciences, Wenzhou University, Wenzhou, 325035, PR China.

^b^ Wenzhou Shanxi Hydro-junction Management Center, Wenzhou, 325200, PR China.

^c^ Wenzhou Wencheng Ecological Environmental Monitoring Station, Wenzhou, China.

* Correspondence:

E-mail addresses: **Yuping Fan**, fanyuping@wzu.edu.cn; **He Zhang**, [zhanghe@wzu.edu.cn](mailto:zhanghe@wzu.edu.cn)

^1^ These authors contributed equally to this work.

**Text S1: Reagents used in this study**

The kits for determining protein concentration, activities of catalase (CAT), and superoxide dismutase (SOD), the contents of reactive oxygen species (ROS) and malondialdehyde (MDA), were purchased from Beyotime (Shanghai, China). The kits for detecting activities of glutathione S-transferase (GST), and acetylcholinesterase (AChE) were obtained from Solarbio (Beijing, China). Phenylmethanesulfonyl fluoride (PMSF), and protease inhibitor cocktails for mammalian cell and tissue extracts were from Beyotime (Shanghai, China). Ethyl 3-aminobenzoate methanesulfonate (MS-222) was purchased from Sigma-Aldrich (St Louis, USA). Total RNA extract reagent TRIzol, and Power SYBR® Green PCR master mixture kits were obtained from Life Technology (NY, USA). The reverse transcription kit PrimeScript™ RT reagent kit with gDNA eraser was purchased from Takara (Liaoning, China).

**Text S2: The details for RCS preparation**

In brief, cyanobacterial cells from the batch cultures at the mid-exponential phase were collected via centrifugation (4 ℃, 30 min, 12, 000 rpm), and then were stored at -80 °C for subsequent studies (Niu et al., 2021). *A.gracile* cells were re-suspended in zebrafish embryo medium at a density of 1.0 × 10^7^ cells/mL, and then broken by an Ultrasonic Processor (Scientz-8TD, Ningbo, China) within an ice bath. The supernatant was filtered through a 0.45 μm filter (Millipore, Merck, Germany) to remove cell debris, and then used for dilution to designated concentration respectively (with embryo medium (EM) (0.294 g/L CaCl_2_, 0.123 g/L MgSO_4_, 0.065 g/L NaHCO_3_, and 0.006 g/L KCl) for the following exposure assays (Niu et al., 2021; Su et al., 2023).

**Text S3: The details for oxidative stress analysis**

Two μL of the obtained supernatant was used for protein concentration determination by a bicinchoninic acid assay (BCA) protein assay kit, and the left was used for detection of activities of CAT, GST, and the content of MDA with corresponding assay kit (Niu et al., 2021), respectively. The absorbance value was measured using a multifunction plate reader (Molecular devices, USA).

The ROS level was determined using DCFH-DA probe (Zhou et al., 2021). In brief, ten zebrafish larvae from each group were incubated with 10 μM of DCFH-DA at 28° C for 30 min in the dark. Then, larvae were washed three times with PBS, and anesthetized with 0.1% of MS-222. Finally, the treated larvae were photographed under a phase-contrast fluorescent microscope (LEICA, Germany).The fluorescence intensity of each larva was quantified using the Image J software (National Institutes of Health, USA)

**Text S4: The details for locomotor behavior analysis**

For the day-night transition (day: night= 14 h: 10 h), and the data of travel distance and move frequency were collected 30-second intervals. The speed threshold for bursting, cruising and freezing swimming is >20 mm/s, 5–20 mm/s, and <5 mm/s. The illumination (~ 150 μmol photons m^-2^ s^-1^) was on at 8:00 a.m., and off at 22:00 p.m. Next, swimming activity was monitored under light or sound stimulation condition. The program was as following: the zebrafish larvae were subject to light (~ 150 μmol photons m^-2^ s^-1^) for 10 cycles (1 min on; 1 min off), totaling 20 min. After a recovery of two hours, the larvae were subject to sound stimulation (440 Hz) for another 10 cycles (1 min on; 1 min off). The high-throughput track system analyzed the data of the day-night behavioral assay every 10 min bins, and every 30 sec bins for the light or sound stimulation as previous studies (Niu et al., 2021; Su et al., 2023).

**Text S5: The details for toxin analysis**

Cyanotoxin was extracted and analyzed by a Waters ACQUITY UPLCH-Class system coupled to a Xevo TQ tandem quadrupole mass spectrometry equipped with an UPLC BEH Amide 100 × 2.1 mm column (Waters, USA) as previous studies described (Chen et al., 2024). Briefly, *A.gracile* CHAB-1039 cells were freeze-dried, and then broken by ultrasonic treatment in acetonitrile–ultrapure water–formic acid solution (90:10:0.1, v/v/v). Cyanobacterial debris were removed by centrifuged (4 °C, 12, 000 rpm, 20 min), and the supernatant was dried by a vacuum evaporator after filtered with a 0.45-μm GHP membrane (PALL, USA). The residue was re-dissolved in ultrapure water, and then toxins were enriched by a solid phase graphitized carbon black SPE cartridges. Finally, toxins were eluted with 5 mL of acetonitrile/water/acetic acid (20:80:1, v/v/v), following by freeze-drying, and re-dissolving for toxin analysis as previous studies described (Ballot et al., 2010; Chen et al., 2024).

**Text S6：The genes and primers for quantitative PCR analysis**

The transcript levels of several apoptosis and neurodevelopment related genes were detected in the present study. The primers are listed in Table S1.

**Table S1 Primers used in the present study.**

| Gene | Forward primer(5′-3′) | Reverse primer(5′-3′) | Accession number |
| --- | --- | --- | --- |
| *nrf2*  *bax*  *bcl2* | GACAAAATCGGCGACAAAAT  GGCTATTTCAACCAGGGTTCC  TCACTCGTTCAGACCCTCAT | TTAGGCCATGTCCACACGTA  TGCGAATCACCAATGCTGT  ACGCTTTCCACGCACAT | NM_182889.1  NM_131562.2  NM_001030253 |
| *caspase-3* | CCGCTGCCCATCACTA | ATCCTTTCACGACCATCT | NM_131877 |
| *caspase-8* | GATGAGAACCTGACAAGCGGTGATG | GCTCATCCAGTCGCAGAATCAGGT | NM_131340 |
| *caspase-9* | AAATACATAGCAAGGCAACC | CACAGGGAATCAAGAAAGG | NM_15288408 |
| *p53* | GGGCAATCAGCGAGCAAA | ACTGACCTTCCTGAGTCTCCA | NM_001271820.1 |
| *α1-tubulin* | GGCTGGAGTTCAGATCGGCAAT | CGCACCTCATCAATGACAGTGG | NM_194388 |
| *gap-43* | GCAGCAGGAAGTGGAGAAGCCA | GGATTCCTCAGCAGCGTCTGGT | NM_131341 |
| *syn2a* | GTGACCATGCCAGCATTTC | TGGTTCTCCATCTTTCACCTT | NM_001002597 |
| *gfap* | CTCTGAGACAAGCGAAGCAGGA | GCCATCTCCTCCTTCAGCATCT | NM_131373 |
| *shha* | GCAAGATAACGCGCAATTCGGAGA | TGCATCTCTGTGTCATGAGCCTGT | NM_131063.3 |
| *elavl3* | AGACAAGATCAGGCCAGAGCTT | TGGTCTGCAGTTTGAGACCGTTGA | NM_131449 |
| *ache* | CATACGCACAATACGCTGCC | TACACAGCACCATGCGAGTT | NM_131846 |
| *neurod1* | GACTTCCTATACCACGAAGGGCATG | CATCATGCTTTCCTCGCTGTATGAC | NM_130978.2 |
| *neurog1* | CATCTCCCAGCCCACCAATAAG | GAGTAGTCACAGCTTGAGGTTTCC | NM_131041.1 |
| *bdnf* | AGGTCCCCGTGACTAATGGT | CGCTTGTCTATTCCTCGGCA | NM_001308648 |
| *manf* | CGAGCTGCAGTGATGCTTTG | TAAAGCCGCTGGAGGAAACC | NM_001076629 |
| *EF1α* | CTGGAGGCCAGCTCAAACAT | ATCAAGAAGAGTAGTACCGCTAGCATTA | NM_131263.1 |

Note: *bax*, encoding the pro-apoptotic Bax protein of the Bcl-2 family (Tsujimoto, 2003; Kratz et al., 2006). *bcl-2*, encoding the Bcl-2 anti-apoptotic protein of the Bcl-2 family(Tsujimoto, 2003; Kratz et al., 2006). *caspase-3*, *-8*, and *-9* encoding the cysteine proteases Caspase-3, -8, and -9, which are important factors for cleaving death substrates (Salvesen and Dixit, 1997; Inohara and Nuñez, 2000). *p53*, encoding a tumor suppressor P53, which plays a crucial role in control of apoptosis (Bates and Vousden, 1996; Cheng et al., 1997). *nrf2*, encoding nuclear factor erythroid 2-related factor 2, which is a member of the cap'n'collar (CNC)-basic-leucine zipper (bZIP) protein family, and plays an important role in the regulation of antioxidant genes and Phase II metabolism in vertebrates (Ishii et al., 2000; Kobayashi et al., 2002). *α1-tubulin*, encoding a neuron-specific microtubule, which is exclusively expressed in the central nervous system, and is responsible for cytoskeletal organization of neurons and architecture of the brain during the early developmental stages of zebrafish embryos (Gulati-Leekha and Goldman, 2006); *gap-43*, encoding GAP43, which plays a crucial role in neurodevelopment and plasticity (Benowitz and Routtenberg, 1997); *syn2a*, encoding Synapsin IIa, which is a modulator of neurotransmitter release and synaptogenesis during synaptic transmission and neurodevelopment (Kao et al., 1998). *manf*, encoding mesencephalic astrocyte-derived neurotrophic factor (MANF), which is a dopaminergic neurotrophic factor with an important role in the genesis and survival of dopaminergic neurons (Chen et al., 2012). *gfap*, encoding glial fibrillary acidic protein (GFAP), which is an intermediate filament protein, and biomarker of astrocytes, and involved in many important processes in the central nervous system (Nielsen and Jorgensen, 2001). *shha*, encoding the secreted signalling molecule sonic hedgehog a, which is crucial in embryonic development, cell differentiation and proliferation, and nervous system regulation (Sasai et al., 2019). *neurog*, encoding a helixloop-helix transcription factor Neurogenin1, regulating neuronal differentiation (Blader et al., 1997). *neurod*, encoding a basic helix-loop-helix transcription factor Neuronal differentiation, promoting differentiation in newborn neurons (Mueller and Wullimann, 2002).

*bdnf*, encoding brain-derived neurotrophic factor, which is an important for neuronal survival, migration, and differentiation during zebrafish embryonic development (Anand and Mondal, 2020). *elavl3*, encoding the ELAV like neuron-specific RNA binding protein 3, also named HuC, which is an early neuronal marker (Kim et al., 1996). ache encoding the hydrolase acetylcholinesterase (AChE), which can degrade the neurotransmitter acetylcholine, regulating neurobehavioral activity (Bertrand et al., 2001; Niu et al., 2021).

Figure S1


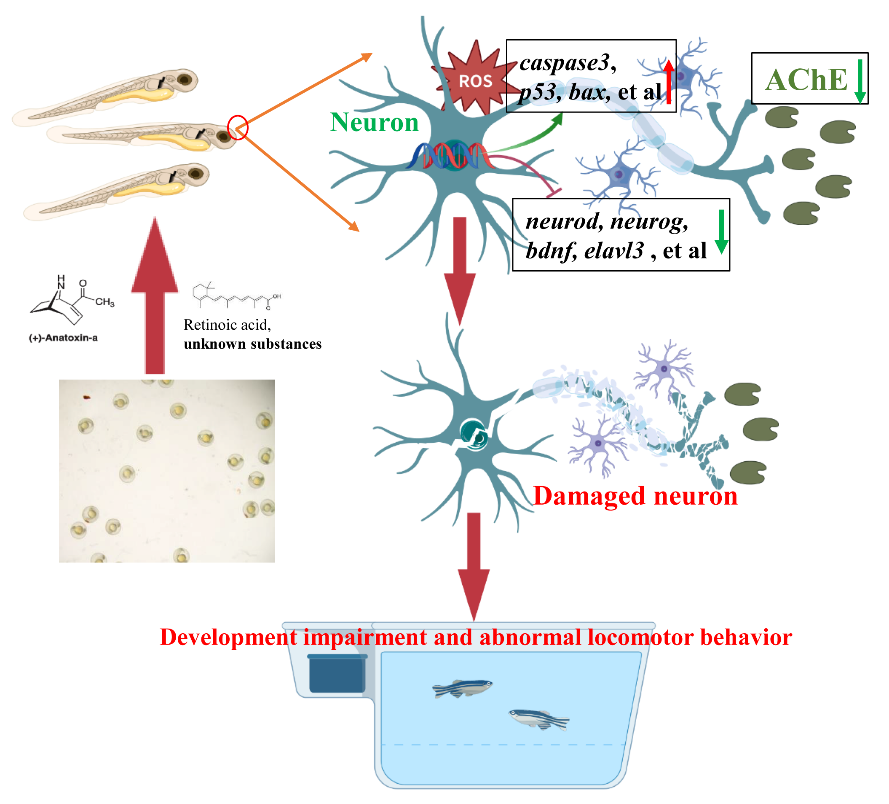


Figure S1. Schematic diagram summarizing the potential mechanism of developmental neurotoxicity in zebrafish larvae exposed to *A. gracile* RCS. The RCS contains ATX-a and other unknown active substances, and they synergistically induce oxidative stress, disrupt neurodevelopment, and activate apoptosis in zebrafish. The green arrows indicates the decrease of gene expression and AChE activity, and the red arrow in the black box indicates the increase of gene expression, and ROS level.

**References**

Anand S.K., Mondal, A.C. (2020). Neuroanatomical distribution and functions of brain-derived neurotrophic factor in zebrafish (*Danio rerio*) brain. *J. Neurosci. Rese*. 98, 754–763. doi:10.1002/jnr.24536

Ballot, A., Fastner, J., Lentz, M., Wiedner, C. (2010). First report of anatoxin-a-producing cyanobacterium *Aphanizomenon issatschenkoi* in northeastern Germany. Toxicon. 56:964-971. doi: 10.1016/j.toxicon.2010.06.021.

Bates, S., Vousden, K.H. *p53* in signaling checkpoint arrest or apoptosis. (1996) .*Curr. Opin. Genet. Dev*. 529 6:12–18. doi:10.1016/s0959-437x(96)90004-0.

Benowitz, L.I., Routtenberg, A. (1997). GAP-43: an intrinsic determinant of neuronal development and plasticity. *Trends. Neurosci*. 20, 84e91. doi:10.1016/s0166-2236(96)10072-2.

Bertrand, C., Chatonnet, A., Takke, C., Yan, Y., Postlethwait, J., Toutant, JP., et al. (2001). Zebrafish acetylcholinesterase is encoded by a single gene localized on linkage group 7. Gene structure and polymorphism; molecular forms and expression pattern during development. *J Biol Chem*. 276:464–74. doi:10.1074/jbc.M006308200.

Blader, P., Fischer, N., Gradwohl, G., Guillemont, F., Strahle, U. (1997). The activity of neurogenin1 is controlled by local cues in the zebrafish embryo. *Development*. 124, 4557–4569. doi:10.1242/dev.124.22.4557.

Chen, Y., Jiang, Y., He, Z., Gao, J., Li, R., Yu, G. (2024). First report of PST-producing *Microseira wollei* from China reveals its novel toxin profile. *Harmful Algae*. 137, 102655. doi: 10.1016/j.hal.2024.102655.

Chen, YC., Sundvik, M., Rozov, S., Priyadarshini, M., Panula, P. (2012). MANF regulates dopaminergic neuron development in larval zebrafish. *Dev. Biol*. 370, 237–249. doi: 10.1016/j.ydbio.2012.07.030.

Cheng, R., Ford, BL., O'Neal, PE., Mathews, CZ., Bradford, CS., Thongtan, T., et al. (1997). Zebrafish (*Danio rerio*) *p53* tumor suppressor gene: cDNA sequence and expression during embryogenesis. *Mol. Mar. Biol. Biotechnol*. 6:88-97.

Gulati-Leekha, A., Goldman, D. (2006). A reporter-assisted mutagenesis screen using alpha 1-tubulin-GFP transgenic zebrafish uncovers missteps during neuronal development and axonogenesis. *Dev. Biol*. 296, 29–47. doi: 10.1016/j.ydbio.2006.03.024.

Inohara, N., Nuñez, G. (2000). Genes with homology to mammalian apoptosis regulators identified in zebrafish. *Cell. Death. Differ*. 7:509-510. doi: 10.1038/sj.cdd.4400679.

Ishii, T., Itoh, K., Takahashi, S., Sato, H., Yanagawa, T., Katoh, Y., et al., (2000). Transcription factor Nrf2 coordinately regulates a group of oxidative stress-inducible genes in macrophages. *J Biol Chem*. 275:16023-9. doi:10.1074/jbc.275.21.16023.

Kao, H. T., Porton, B., Czernik, A. J., Feng, J., Yiu, G., Häring, M., et al. (1998). A third member of the *synapsin* gene family. *Proc. Natl. Acad. Sci.U. S. A*. 95, 4667–4672. doi: 10.1073/pnas.95.8.4667.

Kim, C. H., Ueshima, E., Muraoka, O., Tanaka, H., Yeo, S. Y., Huh, T. L., et al. (1996). Zebrafish *elav/HuC* homologue as a very early neuronal marker. *Neurosci. Lett*. 216, 109–112. doi: 10.1016/0304-3940(96)13021-4.

Kratz, E., Eimon, PM., Mukhyala, K., Stern, H., Zha, J., Strasser A., et al. (2006). Functional characterization of the Bcl-2 gene family in the zebrafish. *Cell. Death. Differ*. 13, 1631-1640. doi:10.1038/sj.cdd.4402016.

Kobayashi, M., Itoh, K., Suzuki, T., Osanai, H., Nishikawa, K., Katoh, Y., et al. (2002). Identification of the interactive interface and phylogenic conservation of the Nrf2-Keap1 system. *Genes. Cells*. 7, 807-820. doi:10.1046/j.1365-2443.2002.00561.x.

Mueller, T., Wullimann, M.F., (2002). Expression domains of *neuroD* (*nrd*) in the early postembryonic zebrafish brain. *Brain. Res. Bull*. 57, 377–379. doi: 10.1016/s0361-9230(01)00694-3.

Nielsen, AL., Jorgensen, AL. (2003). Structural and functional characterization of the zebrafish gene for glial fibrillary acidic protein, GFAP. *Gene.* 310, 123–132. doi:10.1016/s0378-1119(03)00526-2.

Niu, X., Xu, S., Yang, Q., Xu, X., Zheng, M., Li, X., et al. (2021). Toxic effects of the dinoflagellate *Karenia mikimotoi* on zebrafish (*Danio rerio*) larval behavior. *Harmful. Algae*. 103:101996. doi: 10.1016/j.hal.2021.101996.

Salvesen, G.S., Dixit, V.M., (1997). Caspases: intracellular signaling by proteolysis. *Cell*. 91, 443–446. doi:10.1016/s0092-8674(00)80430-4.

Tsujimoto Y. (2003). Cell death regulation by the Bcl-2 protein family in the mitochondria*. J Cell Physiol*. 195, 158-167. doi:10.1002/jcp.10254.

Sasai, N., Toriyama, M., Kondo, T. (2019). Hedgehog signal and genetic disorders. *Front. Genet.* 10, 1103. doi:10.3389/fgene.2019.01103

Su, Z., Guan, K., Liu, Y., Zhang, H., Huang, Z., Zheng, M., et al. (2023). Developmental and behavioral toxicity assessment of opicapone in zebrafish embryos. *Ecotoxicol. Environ. Saf*. 249, 114340. doi: 10.1016/j.ecoenv.2022.114340.

Zhou, W., Li, X., Wang, Y., Wang, J., Zhang, J., Wei, H., et al. (2021). Physiological and transcriptomic changes of zebrafish (*Danio rerio*) embryos-larvae in response to 2-MIB exposure. *J. Hazard. Mater*. 416, 126142. doi: 10.1016/j.jhazmat.2021.126142.
